# Supplementary material for: Enrichment, Characterization, and Proteomic Profiling of Small Extracellular Vesicles Derived from Human Limbal Mesenchymal Stromal Cells and Melanocytes
Source: Cells. 2024 Apr 4;13(7):623. doi: 10.3390/cells13070623 (PMC11011788; doi:10.3390/cells13070623)
Supplement: Supplementary file 1 [file cells-13-00623-s001.zip › Supplementary File S1.pptx]

## Slide 1
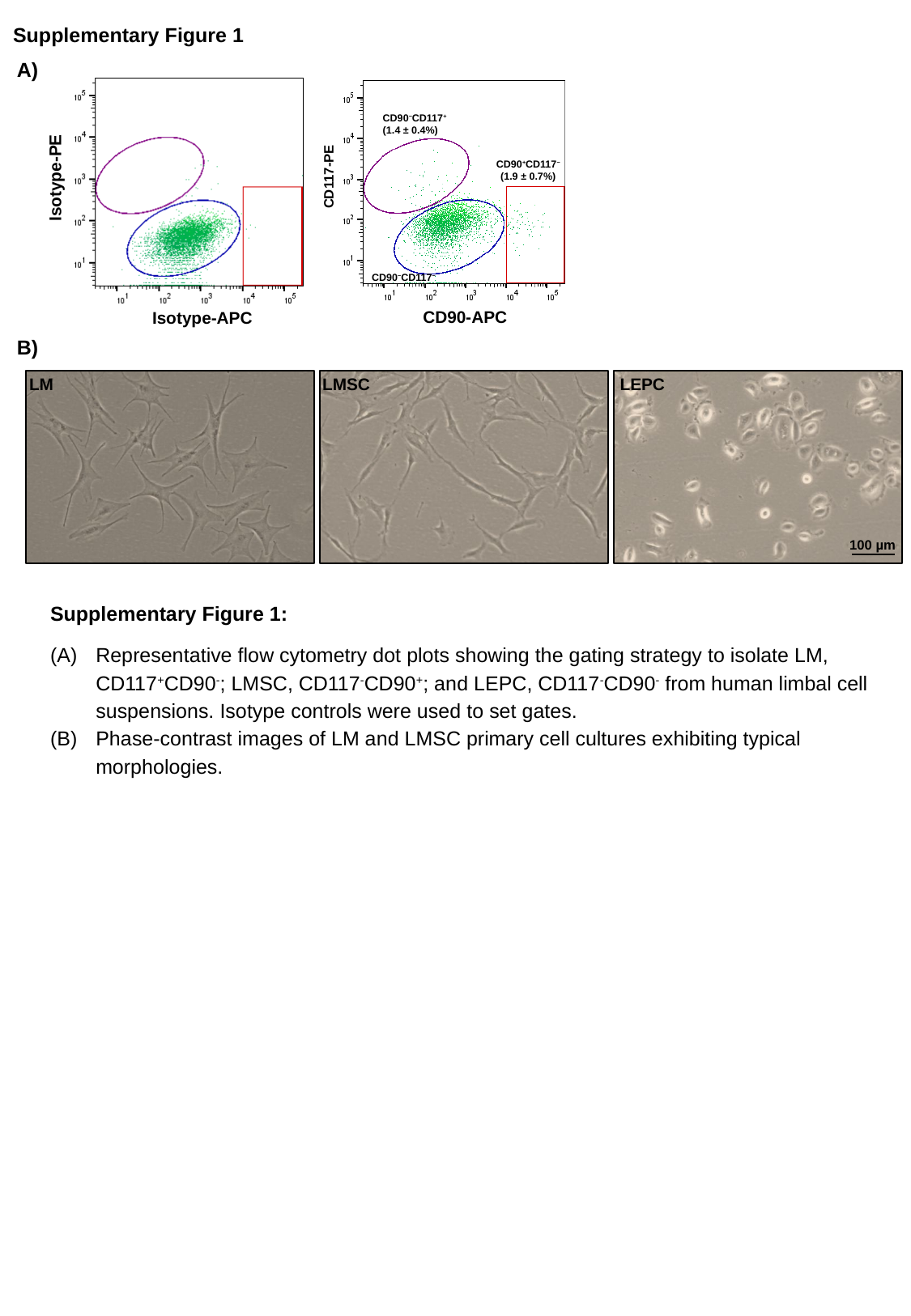

Supplementary Figure 1
A)
CD90−CD117+
(1.4 ± 0.4%)
CD90+CD117−
(1.9 ± 0.7%)
Isotype-PE
CD117-PE
CD90−CD117−
CD90-APC
Isotype-APC
B)
LEPC
LMSC
LM
100 µm
Supplementary Figure 1:
Representative flow cytometry dot plots showing the gating strategy to isolate LM, CD117+CD90-; LMSC, CD117-CD90+; and LEPC, CD117-CD90- from human limbal cell suspensions. Isotype controls were used to set gates.
Phase-contrast images of LM and LMSC primary cell cultures exhibiting typical morphologies.

## Slide 2
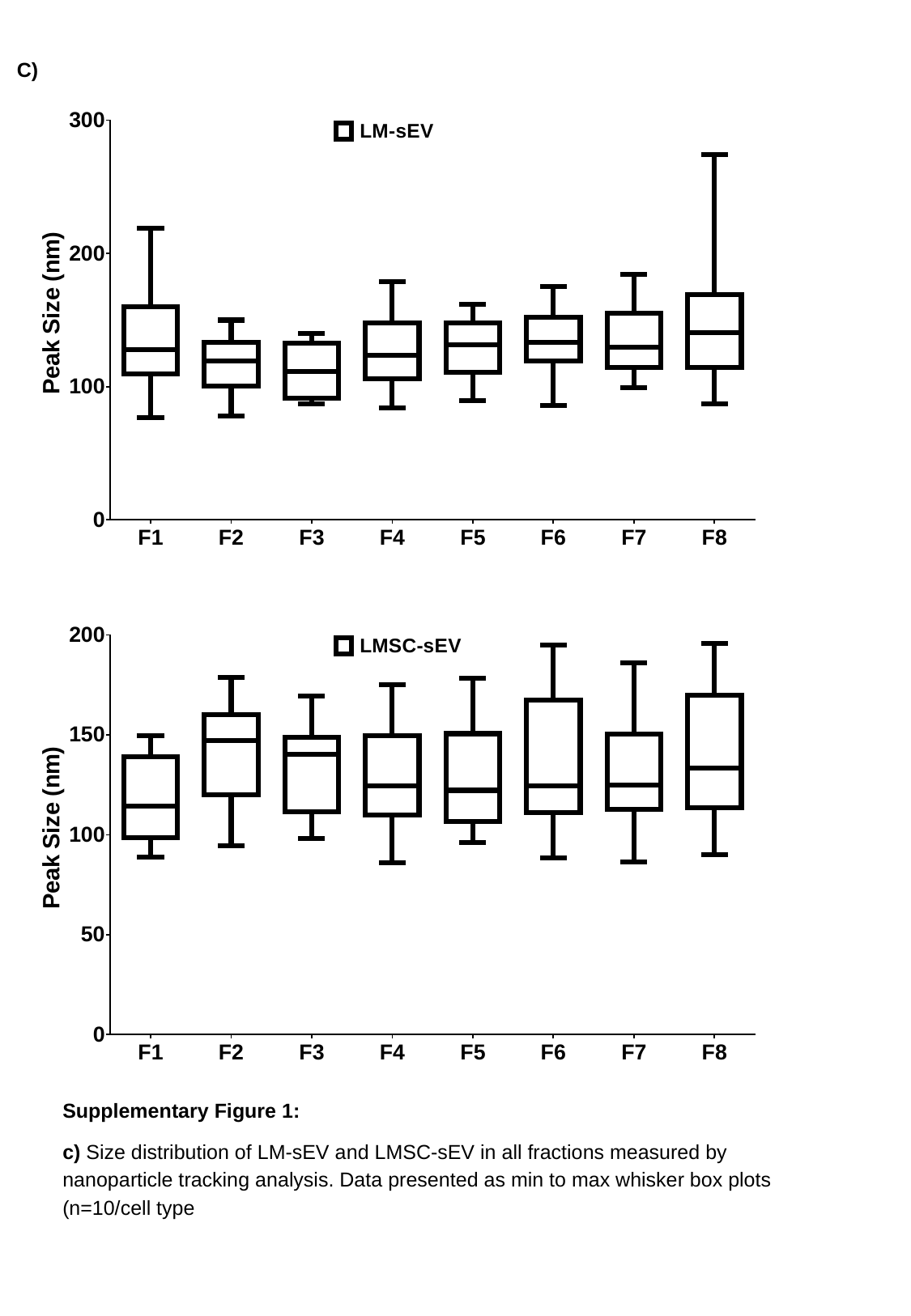

C)
Supplementary Figure 1:
c) Size distribution of LM-sEV and LMSC-sEV in all fractions measured by nanoparticle tracking analysis. Data presented as min to max whisker box plots (n=10/cell type
