# Supplementary material for: Enrichment, Characterization, and Proteomic Profiling of Small Extracellular Vesicles Derived from Human Limbal Mesenchymal Stromal Cells and Melanocytes
Source: Cells. 2024 Apr 4;13(7):623. doi: 10.3390/cells13070623 (PMC11011788; doi:10.3390/cells13070623)
Supplement: Supplementary file 1 [file cells-13-00623-s001.zip › Supplementary File S2.pptx]

## Slide 1
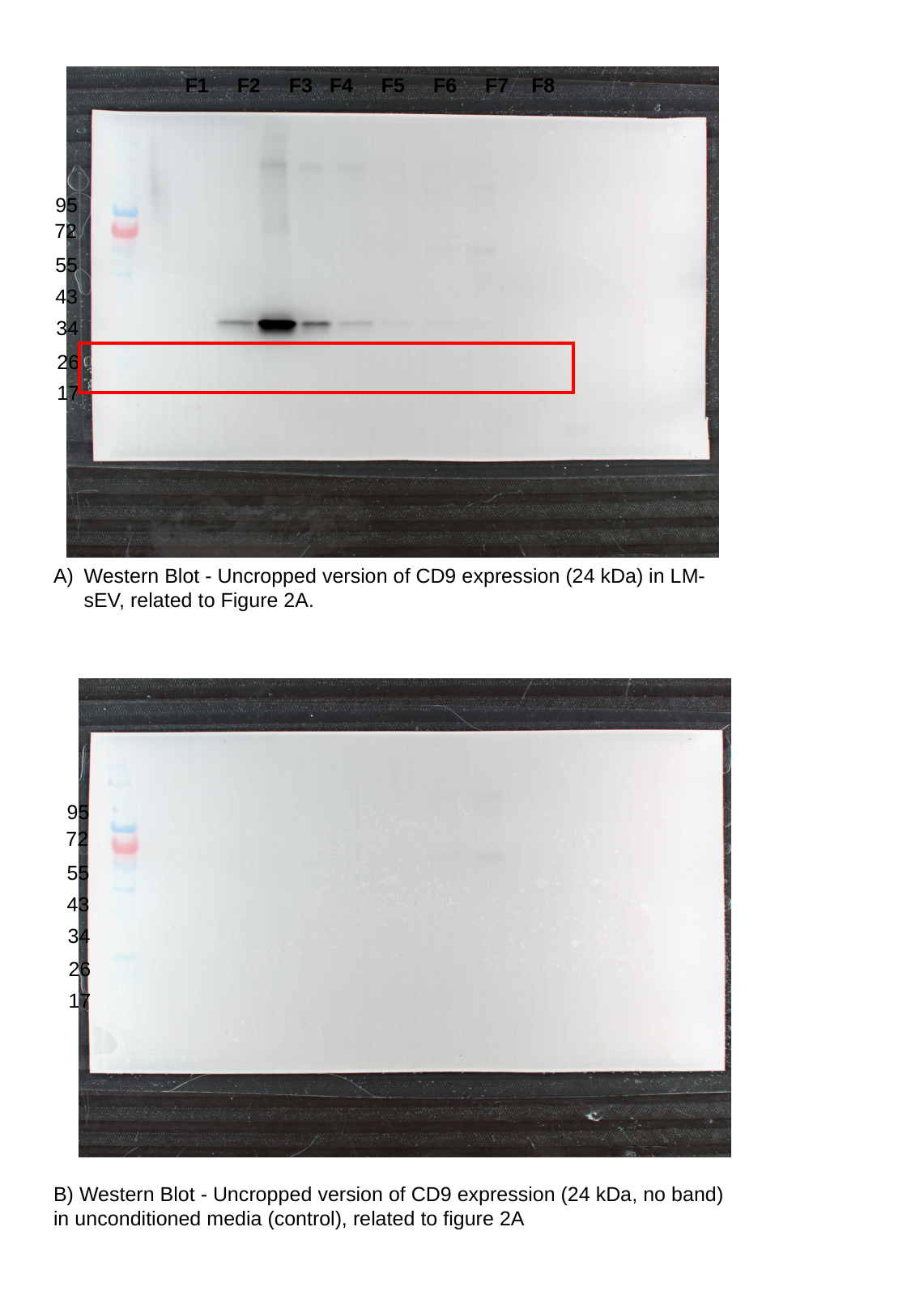

F1 F2 F3 F4 F5 F6 F7 F8
95
72
55
43
34
26
17
Western Blot - Uncropped version of CD9 expression (24 kDa) in LM-sEV, related to Figure 2A.
95
72
55
43
34
26
17
B) Western Blot - Uncropped version of CD9 expression (24 kDa, no band) in unconditioned media (control), related to figure 2A

## Slide 2
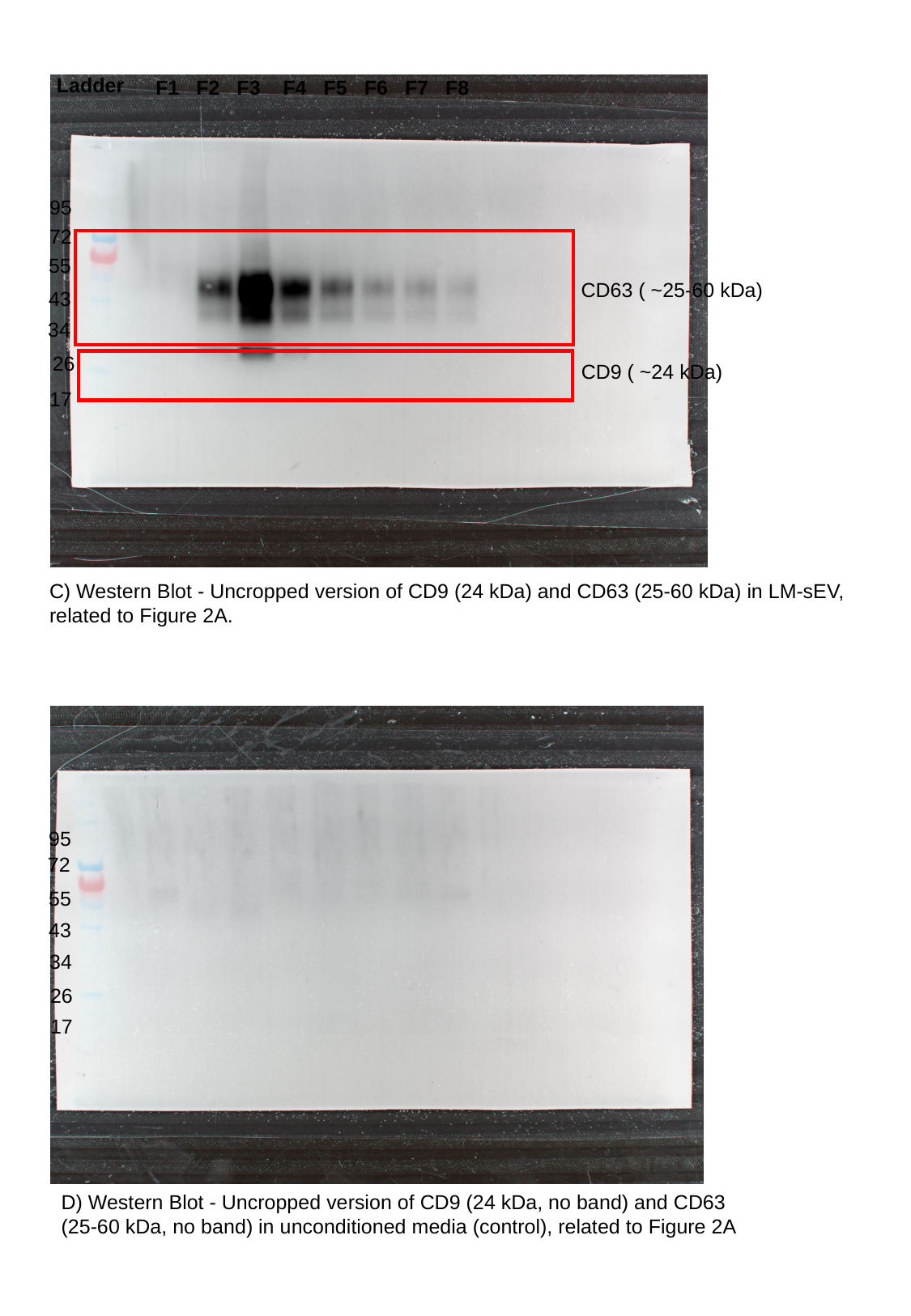

Ladder
 F1 F2 F3 F4 F5 F6 F7 F8
95
72
55
CD63 ( ~25-60 kDa)
43
34
26
CD9 ( ~24 kDa)
17
C) Western Blot - Uncropped version of CD9 (24 kDa) and CD63 (25-60 kDa) in LM-sEV,
related to Figure 2A.
95
72
55
43
34
26
17
D) Western Blot - Uncropped version of CD9 (24 kDa, no band) and CD63
(25-60 kDa, no band) in unconditioned media (control), related to Figure 2A

## Slide 3
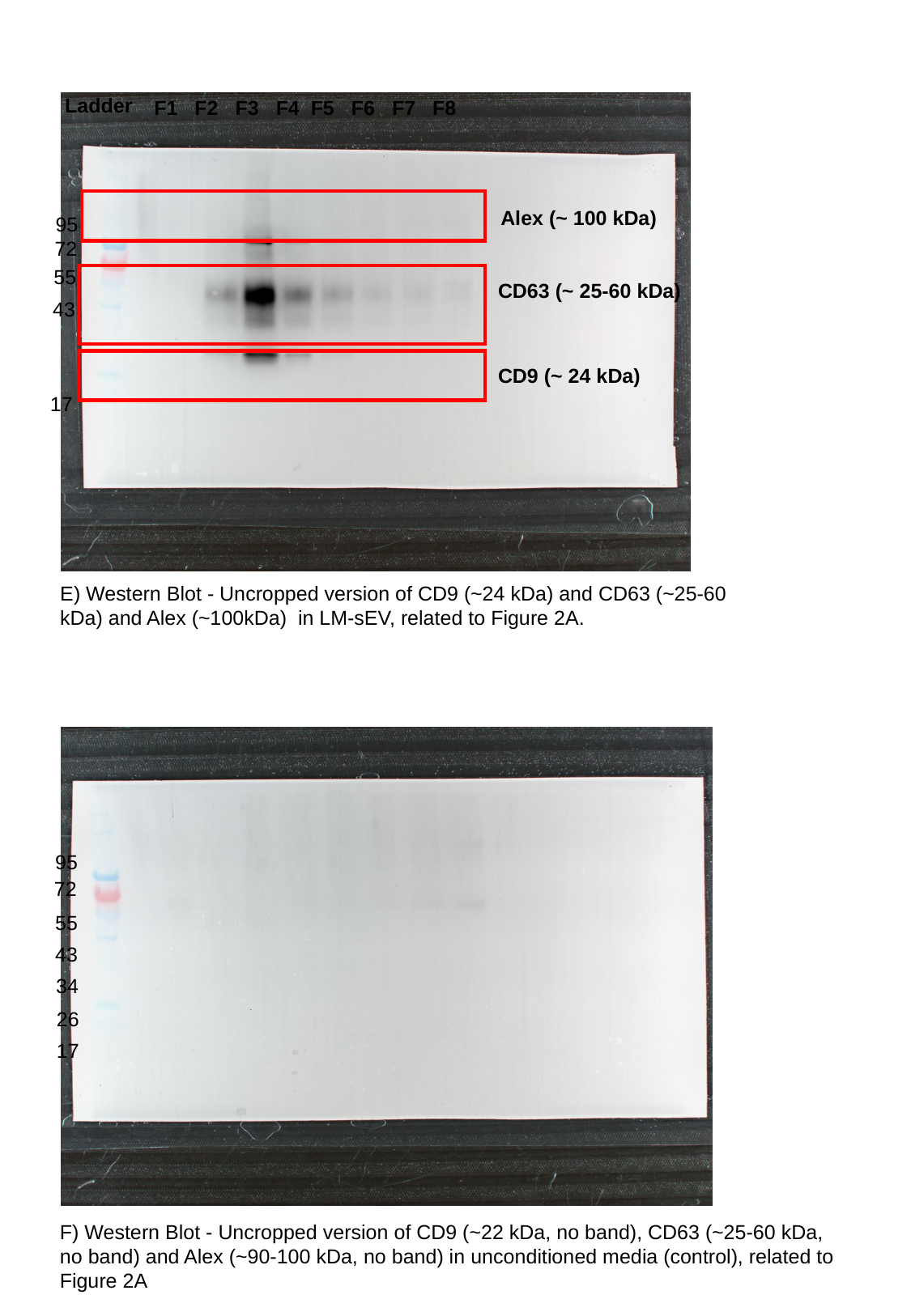

Ladder
 F1 F2 F3 F4 F5 F6 F7 F8
Alex (~ 100 kDa)
95
72
55
CD63 (~ 25-60 kDa)
43
CD9 (~ 24 kDa)
17
E) Western Blot - Uncropped version of CD9 (~24 kDa) and CD63 (~25-60 kDa) and Alex (~100kDa) in LM-sEV, related to Figure 2A.
95
72
55
43
34
26
17
F) Western Blot - Uncropped version of CD9 (~22 kDa, no band), CD63 (~25-60 kDa, no band) and Alex (~90-100 kDa, no band) in unconditioned media (control), related to Figure 2A

## Slide 4
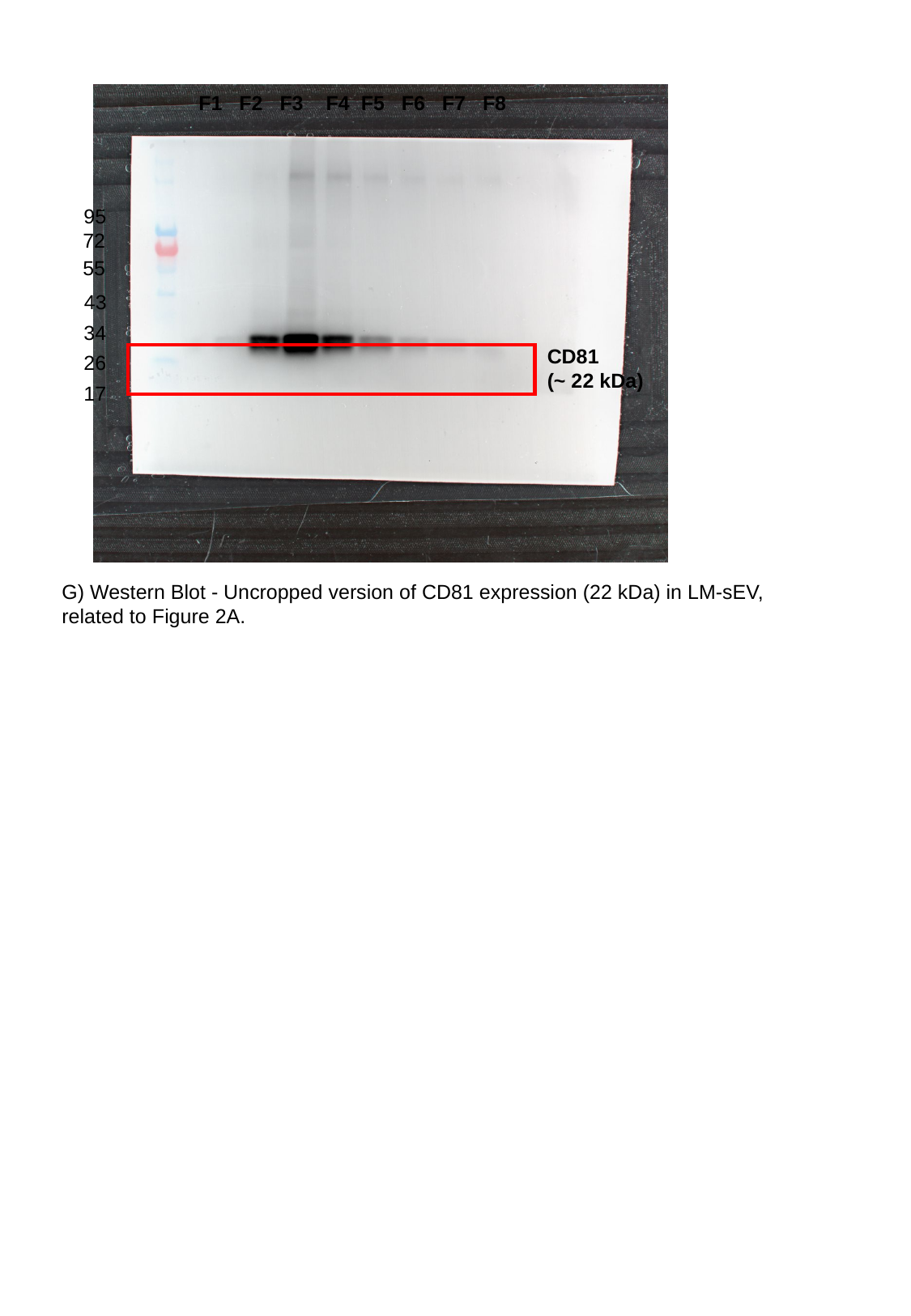

F1 F2 F3 F4 F5 F6 F7 F8
95
72
55
43
34
CD81
(~ 22 kDa)
26
17
G) Western Blot - Uncropped version of CD81 expression (22 kDa) in LM-sEV, related to Figure 2A.

## Slide 5
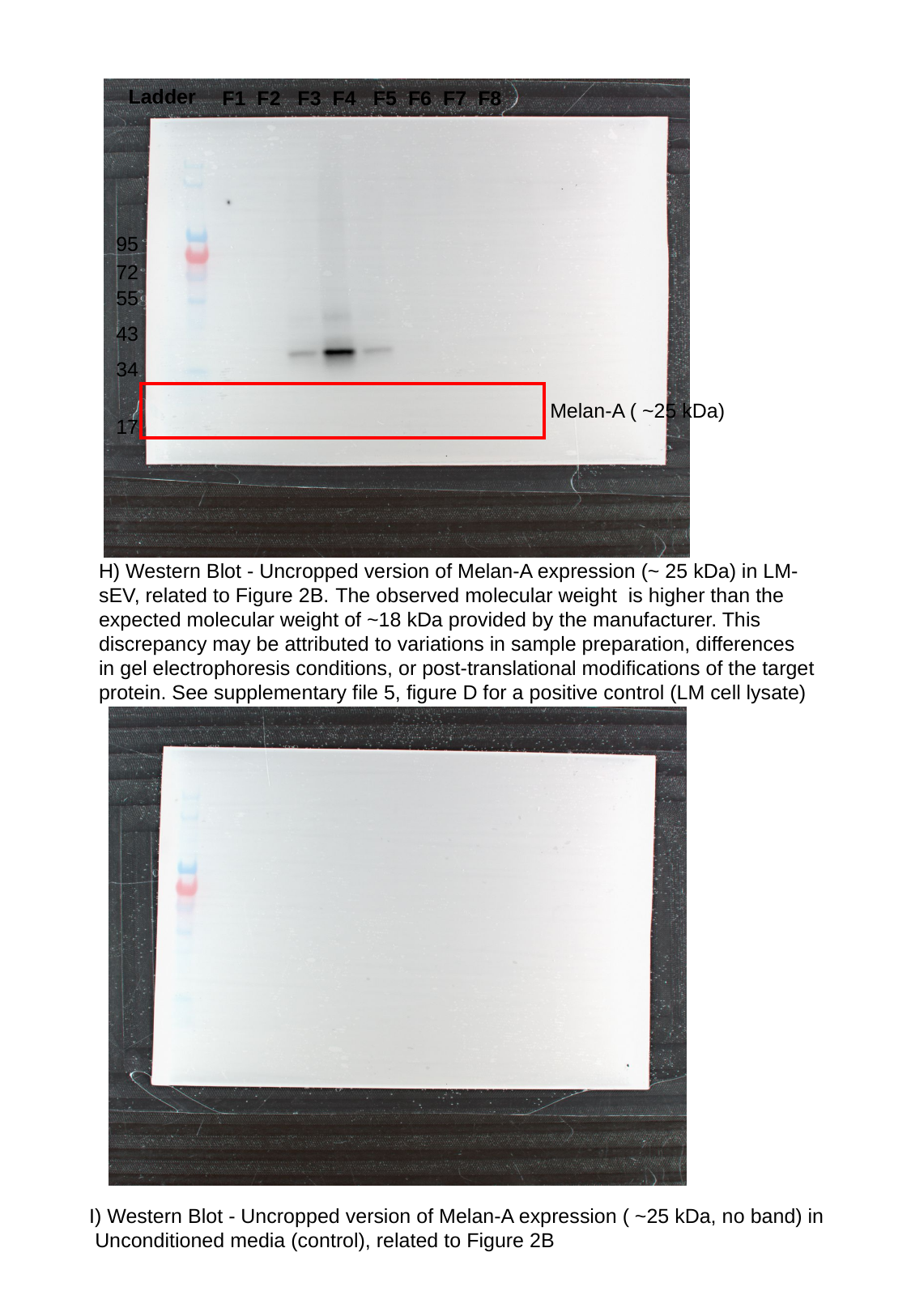

Ladder
 F1 F2 F3 F4 F5 F6 F7 F8
95
72
55
43
34
Melan-A ( ~25 kDa)
17
H) Western Blot - Uncropped version of Melan-A expression (~ 25 kDa) in LM-sEV, related to Figure 2B. The observed molecular weight is higher than the expected molecular weight of ~18 kDa provided by the manufacturer. This discrepancy may be attributed to variations in sample preparation, differences in gel electrophoresis conditions, or post-translational modifications of the target protein. See supplementary file 5, figure D for a positive control (LM cell lysate)
I) Western Blot - Uncropped version of Melan-A expression ( ~25 kDa, no band) in
 Unconditioned media (control), related to Figure 2B
