# Supplementary material for: Enrichment, Characterization, and Proteomic Profiling of Small Extracellular Vesicles Derived from Human Limbal Mesenchymal Stromal Cells and Melanocytes
Source: Cells. 2024 Apr 4;13(7):623. doi: 10.3390/cells13070623 (PMC11011788; doi:10.3390/cells13070623)
Supplement: Supplementary file 1 [file cells-13-00623-s001.zip › Supplementary File S3.pptx]

## Slide 1
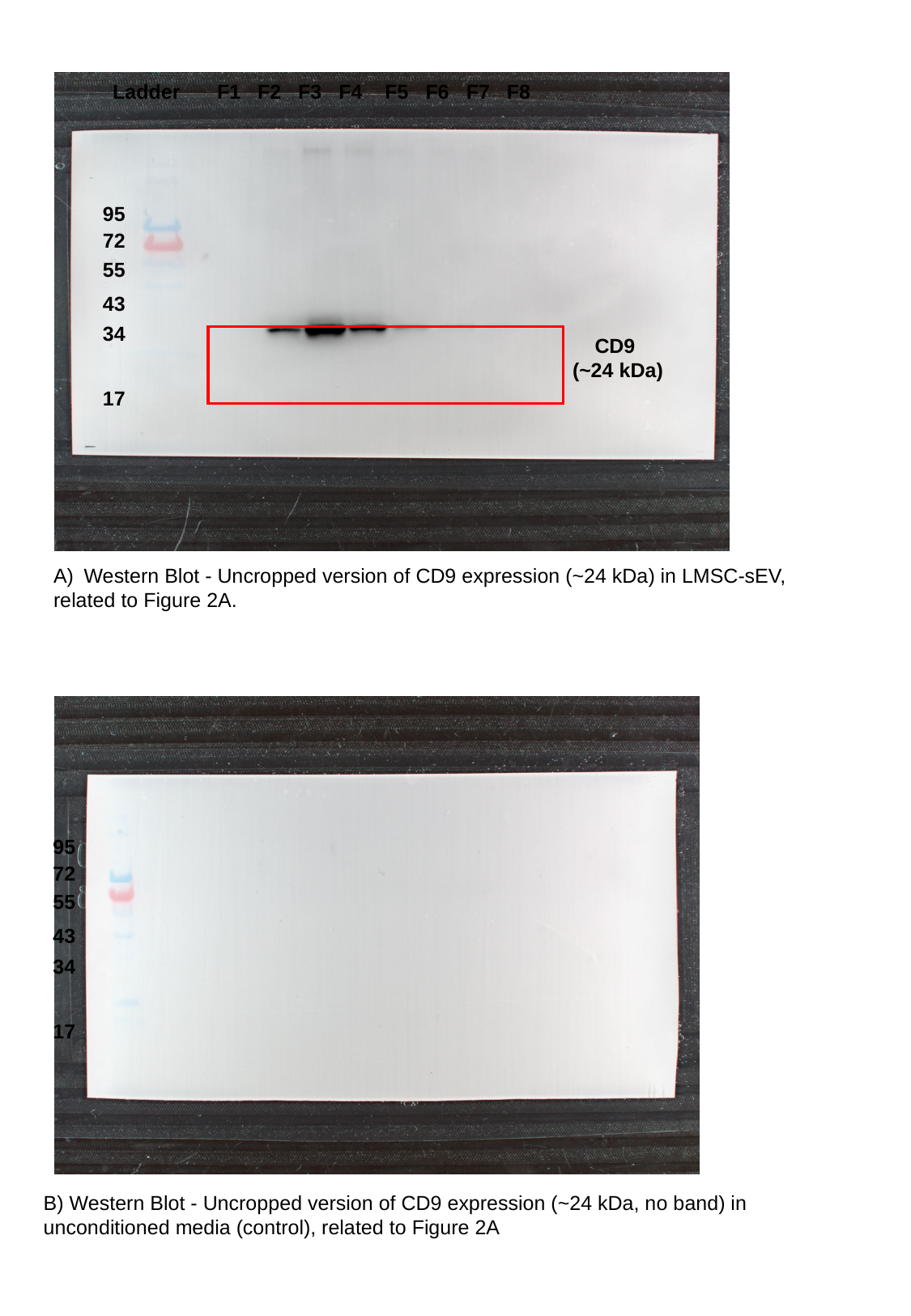

Ladder
 F1 F2 F3 F4 F5 F6 F7 F8
95
72
55
43
34
CD9
(~24 kDa)
17
Western Blot - Uncropped version of CD9 expression (~24 kDa) in LMSC-sEV,
related to Figure 2A.
95
72
55
43
34
17
B) Western Blot - Uncropped version of CD9 expression (~24 kDa, no band) in
unconditioned media (control), related to Figure 2A

## Slide 2
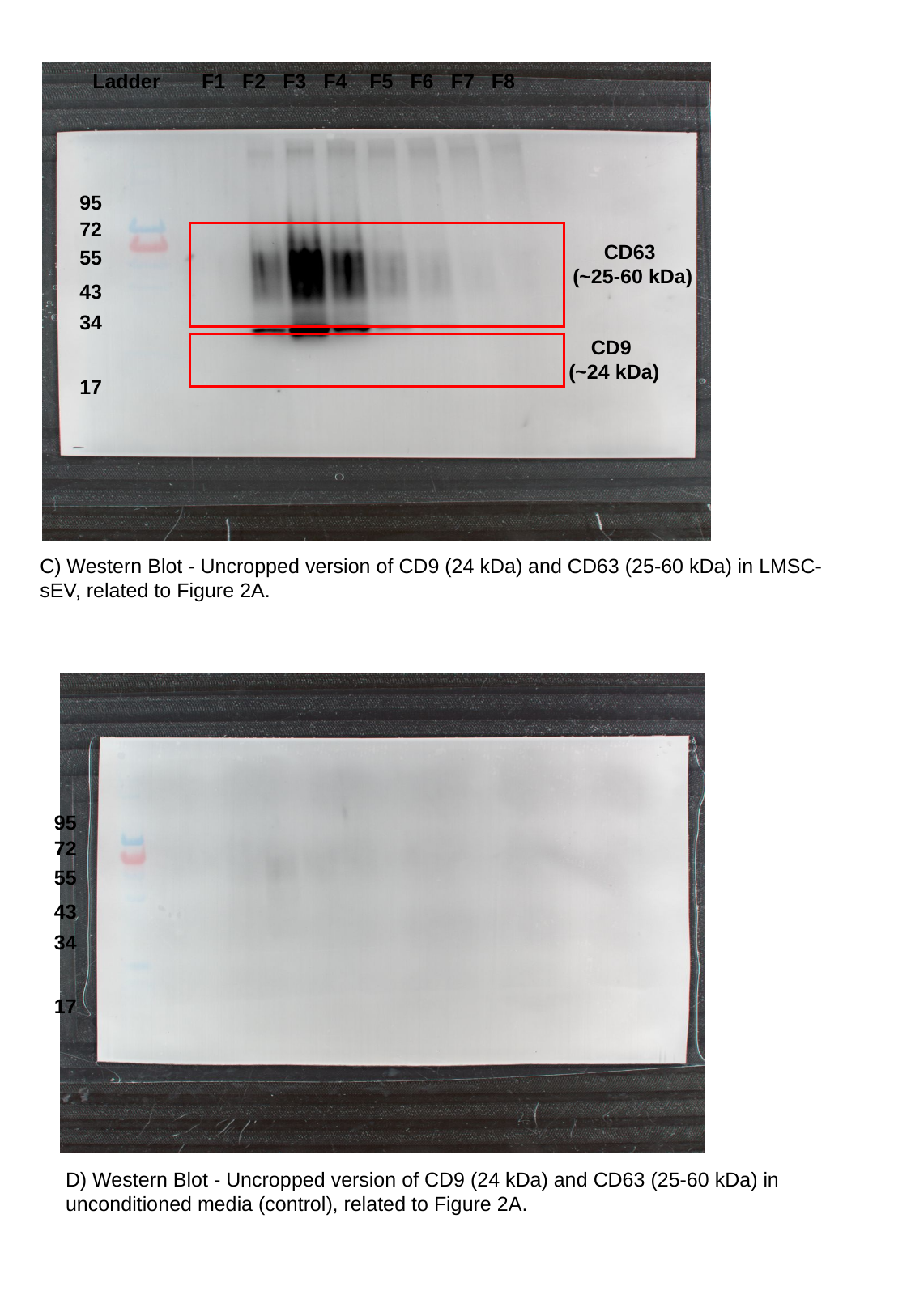

Ladder
 F1 F2 F3 F4 F5 F6 F7 F8
95
72
CD63
(~25-60 kDa)
55
43
34
CD9
(~24 kDa)
17
C) Western Blot - Uncropped version of CD9 (24 kDa) and CD63 (25-60 kDa) in LMSC-sEV, related to Figure 2A.
95
72
55
43
34
17
D) Western Blot - Uncropped version of CD9 (24 kDa) and CD63 (25-60 kDa) in
unconditioned media (control), related to Figure 2A.

## Slide 3
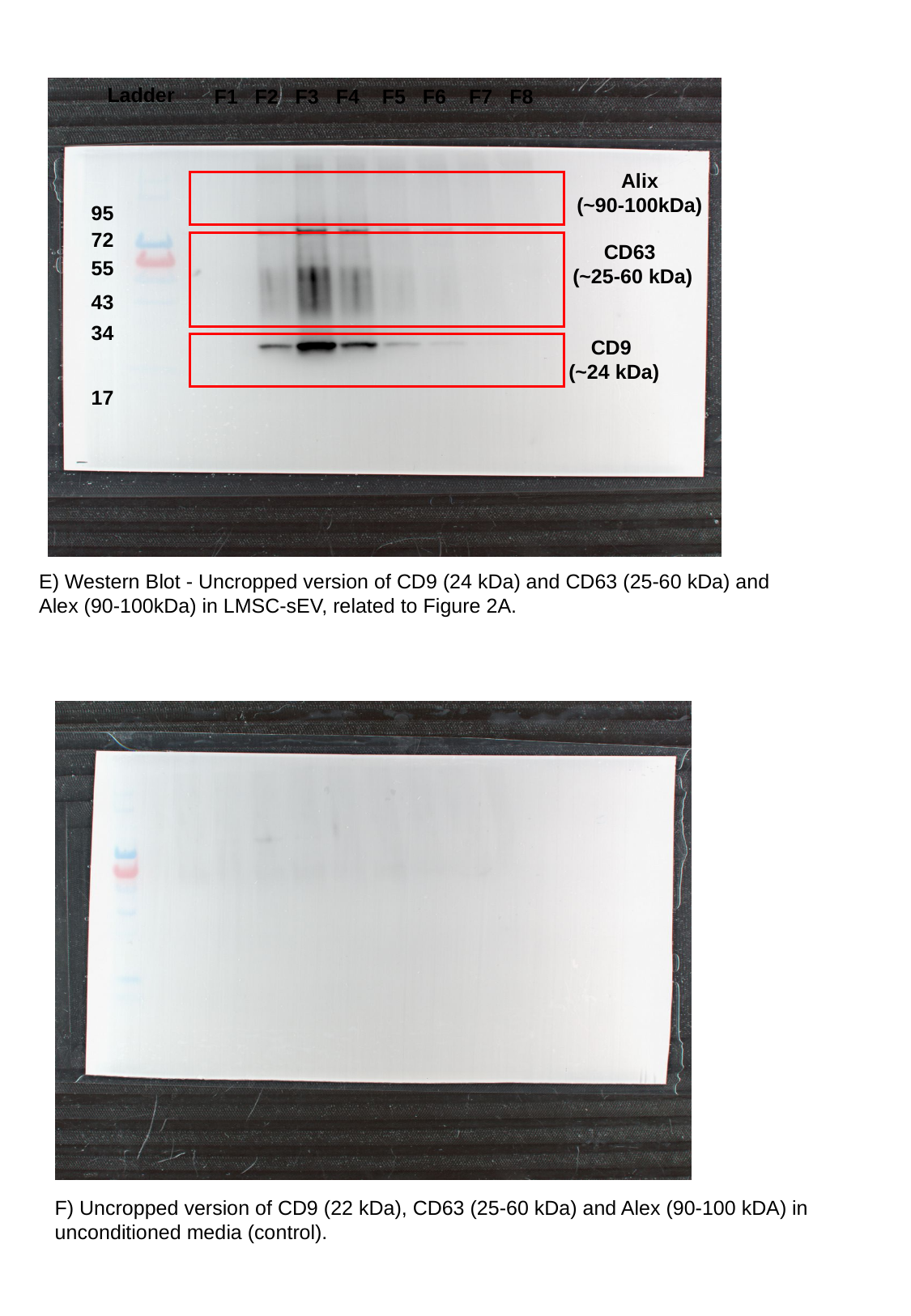

Ladder
 F1 F2 F3 F4 F5 F6 F7 F8
Alix
(~90-100kDa)
95
72
CD63
(~25-60 kDa)
55
43
34
CD9
(~24 kDa)
17
E) Western Blot - Uncropped version of CD9 (24 kDa) and CD63 (25-60 kDa) and Alex (90-100kDa) in LMSC-sEV, related to Figure 2A.
F) Uncropped version of CD9 (22 kDa), CD63 (25-60 kDa) and Alex (90-100 kDA) in
unconditioned media (control).

## Slide 4
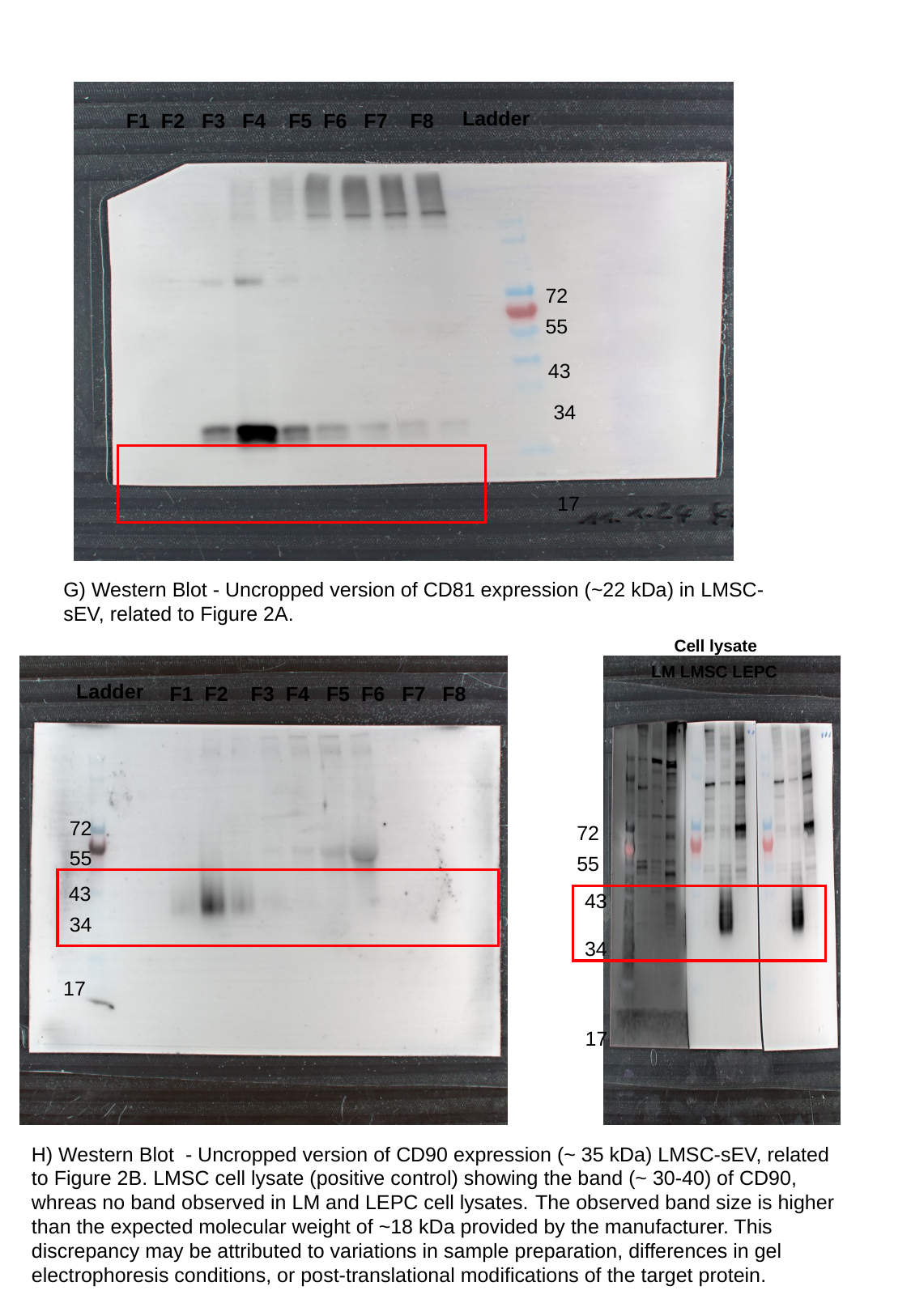

Ladder
 F1 F2 F3 F4 F5 F6 F7 F8
72
55
43
34
17
G) Western Blot - Uncropped version of CD81 expression (~22 kDa) in LMSC-sEV, related to Figure 2A.
Cell lysate
 LM LMSC LEPC
Ladder
 F1 F2 F3 F4 F5 F6 F7 F8
72
72
55
55
43
43
34
34
17
17
H) Western Blot - Uncropped version of CD90 expression (~ 35 kDa) LMSC-sEV, related to Figure 2B. LMSC cell lysate (positive control) showing the band (~ 30-40) of CD90, whreas no band observed in LM and LEPC cell lysates. The observed band size is higher than the expected molecular weight of ~18 kDa provided by the manufacturer. This discrepancy may be attributed to variations in sample preparation, differences in gel electrophoresis conditions, or post-translational modifications of the target protein.
