# Supplementary material for: Enrichment, Characterization, and Proteomic Profiling of Small Extracellular Vesicles Derived from Human Limbal Mesenchymal Stromal Cells and Melanocytes
Source: Cells. 2024 Apr 4;13(7):623. doi: 10.3390/cells13070623 (PMC11011788; doi:10.3390/cells13070623)
Supplement: Supplementary file 1 [file cells-13-00623-s001.zip › Supplementary File S4.pptx]

## Slide 1
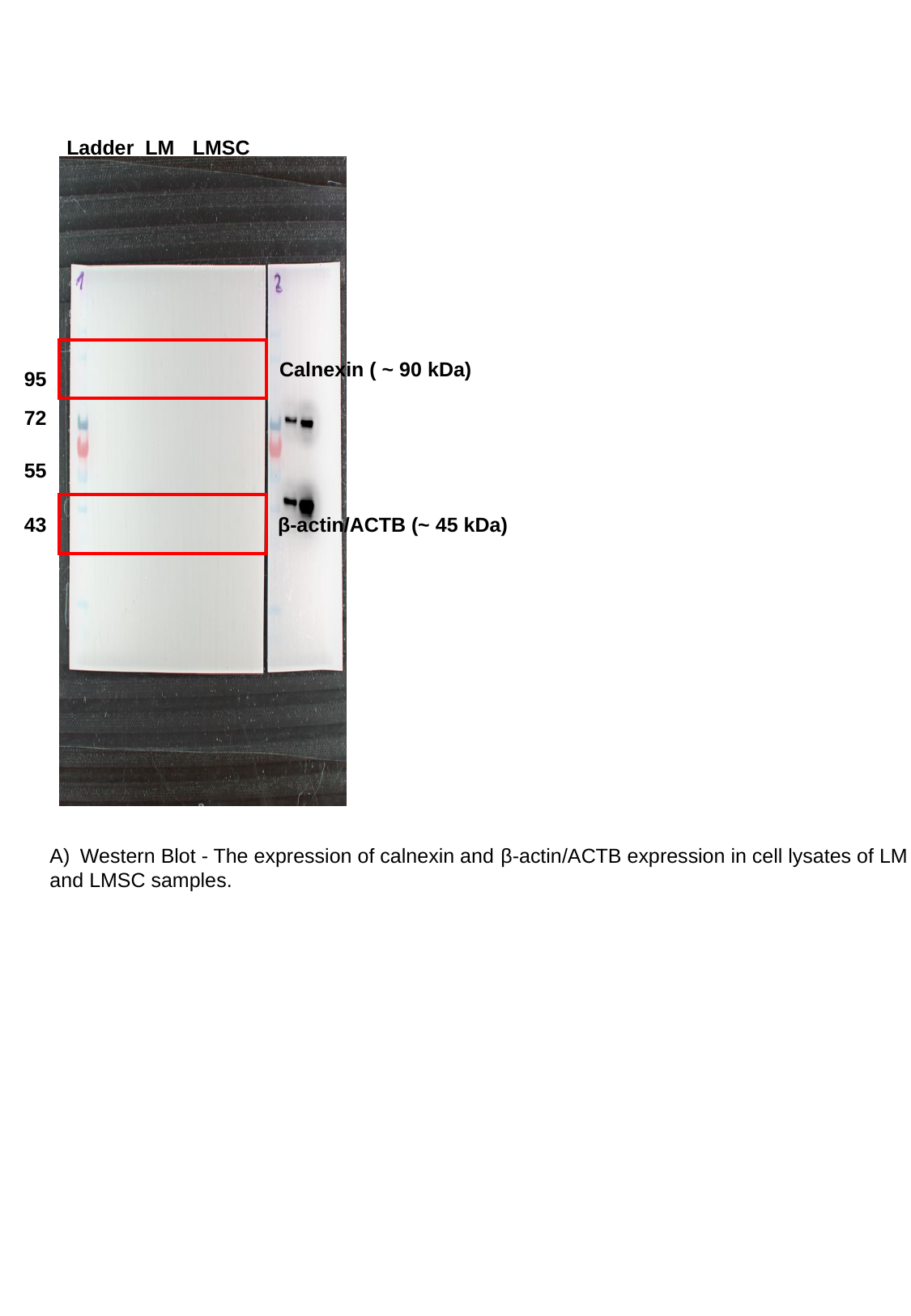

Ladder
LM
LMSC
Calnexin ( ~ 90 kDa)
95
72
55
β-actin/ACTB (~ 45 kDa)
43
Western Blot - The expression of calnexin and β-actin/ACTB expression in cell lysates of LM
and LMSC samples.

## Slide 2
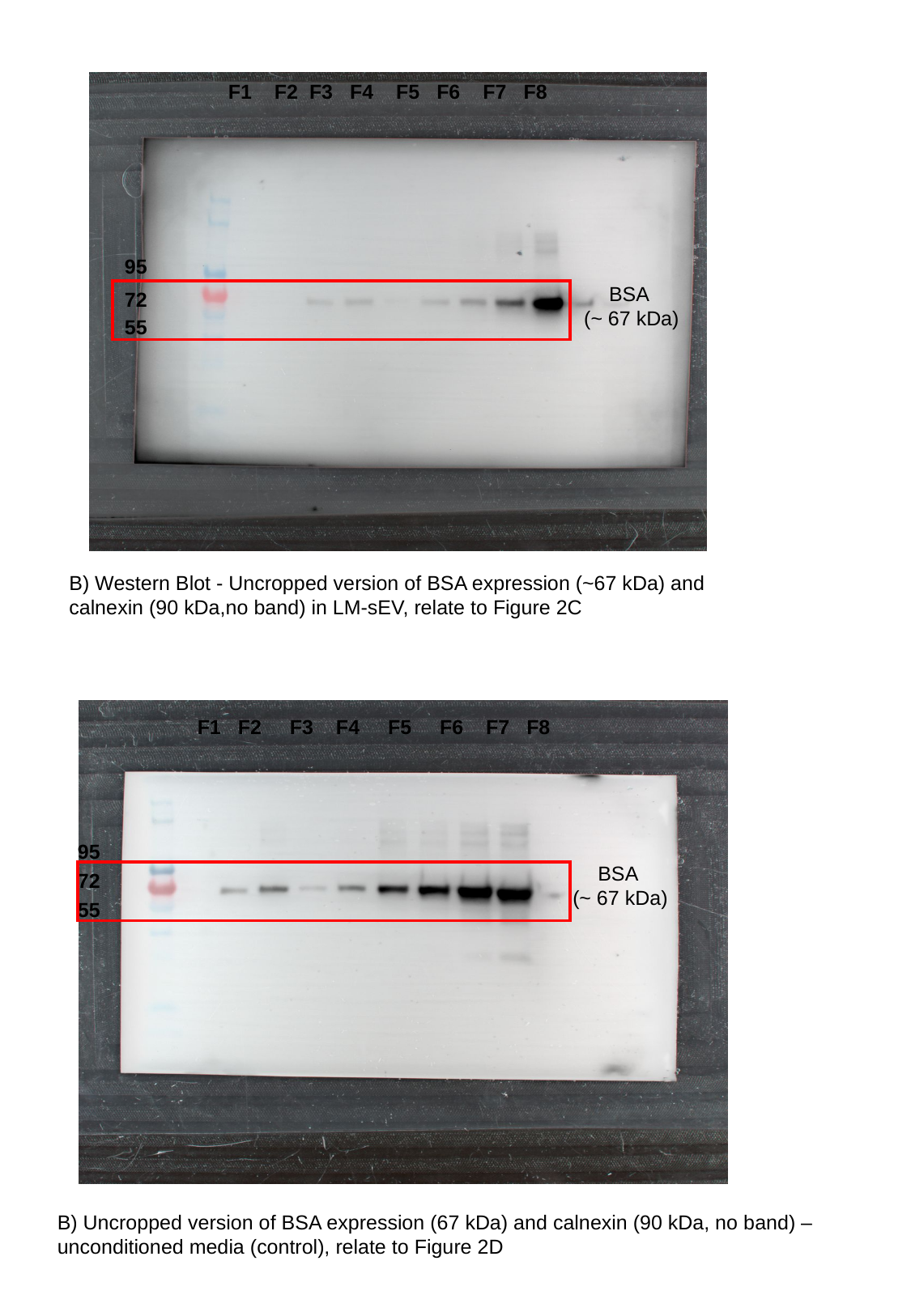

F1 F2 F3 F4 F5 F6 F7 F8
95
BSA
(~ 67 kDa)
72
55
B) Western Blot - Uncropped version of BSA expression (~67 kDa) and calnexin (90 kDa,no band) in LM-sEV, relate to Figure 2C
 F1 F2 F3 F4 F5 F6 F7 F8
95
BSA
(~ 67 kDa)
72
55
B) Uncropped version of BSA expression (67 kDa) and calnexin (90 kDa, no band) –
unconditioned media (control), relate to Figure 2D

## Slide 3
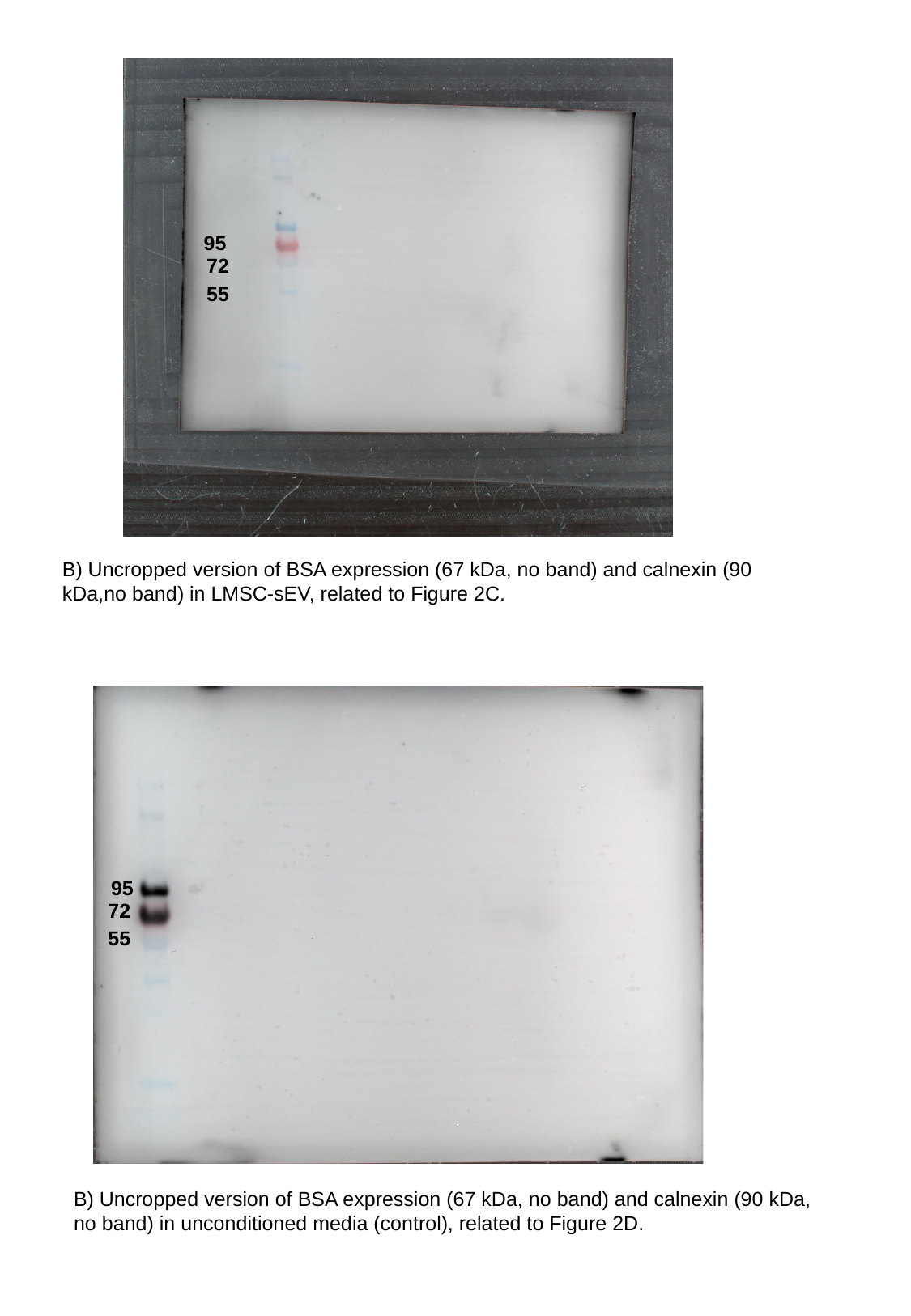

95
72
55
B) Uncropped version of BSA expression (67 kDa, no band) and calnexin (90 kDa,no band) in LMSC-sEV, related to Figure 2C.
 95
72
55
B) Uncropped version of BSA expression (67 kDa, no band) and calnexin (90 kDa, no band) in unconditioned media (control), related to Figure 2D.
