# Supplementary material for: Enrichment, Characterization, and Proteomic Profiling of Small Extracellular Vesicles Derived from Human Limbal Mesenchymal Stromal Cells and Melanocytes
Source: Cells. 2024 Apr 4;13(7):623. doi: 10.3390/cells13070623 (PMC11011788; doi:10.3390/cells13070623)
Supplement: Supplementary file 1 [file cells-13-00623-s001.zip › Supplementary File S5.pptx]

## Slide 1
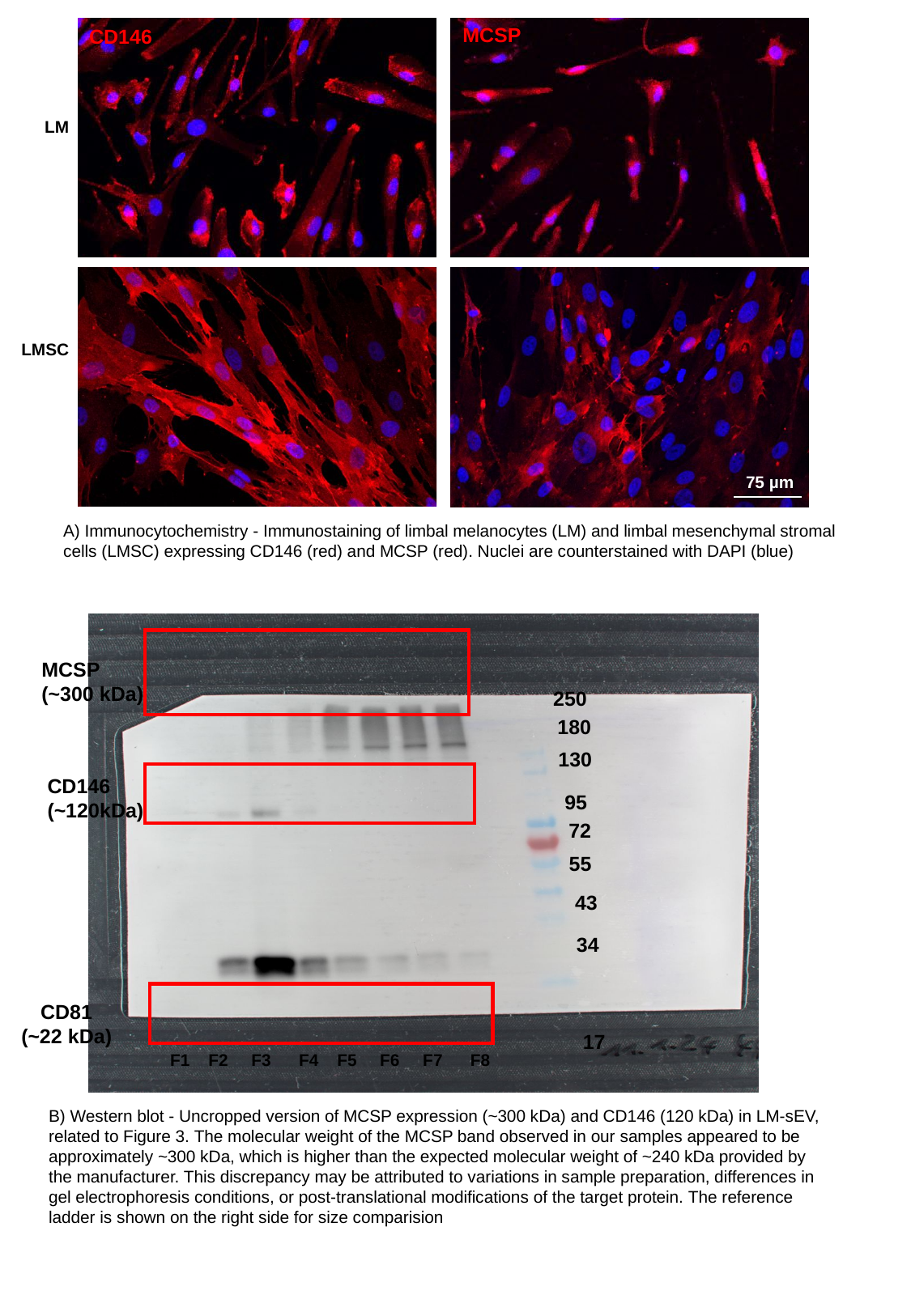

MCSP
CD146
LM
LMSC
75 µm
A) Immunocytochemistry - Immunostaining of limbal melanocytes (LM) and limbal mesenchymal stromal cells (LMSC) expressing CD146 (red) and MCSP (red). Nuclei are counterstained with DAPI (blue)
MCSP
(~300 kDa)
250
180
130
CD146
(~120kDa)
95
72
55
43
34
CD81
(~22 kDa)
17
 F1 F2 F3 F4 F5 F6 F7 F8
B) Western blot - Uncropped version of MCSP expression (~300 kDa) and CD146 (120 kDa) in LM-sEV, related to Figure 3. The molecular weight of the MCSP band observed in our samples appeared to be approximately ~300 kDa, which is higher than the expected molecular weight of ~240 kDa provided by the manufacturer. This discrepancy may be attributed to variations in sample preparation, differences in gel electrophoresis conditions, or post-translational modifications of the target protein. The reference ladder is shown on the right side for size comparision

## Slide 2
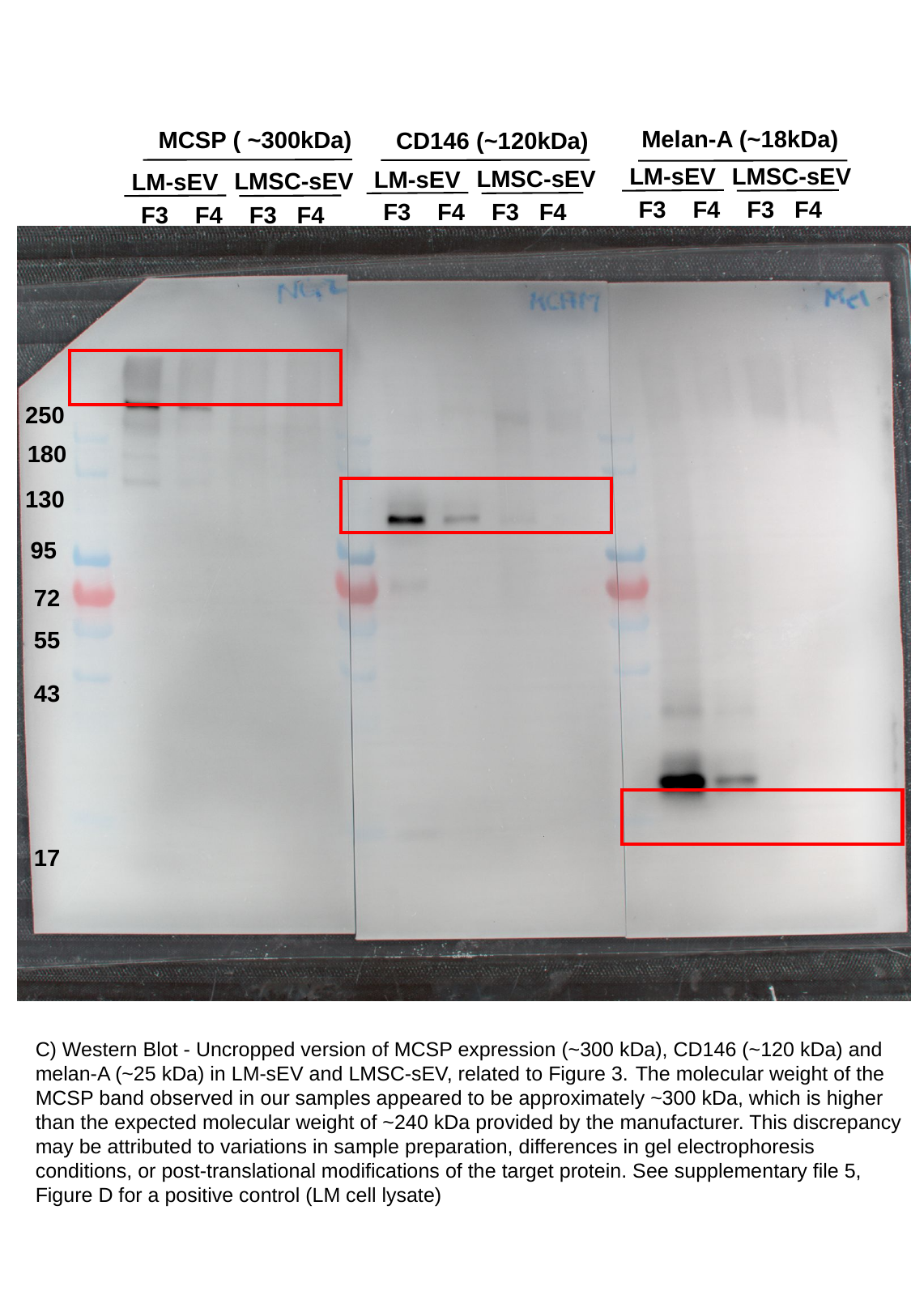

Melan-A (~18kDa)
MCSP ( ~300kDa)
CD146 (~120kDa)
 LMSC-sEV
LM-sEV
 LMSC-sEV
LM-sEV
 LMSC-sEV
LM-sEV
 F3 F4 F3 F4
 F3 F4 F3 F4
 F3 F4 F3 F4
250
180
130
95
72
55
43
17
C) Western Blot - Uncropped version of MCSP expression (~300 kDa), CD146 (~120 kDa) and melan-A (~25 kDa) in LM-sEV and LMSC-sEV, related to Figure 3. The molecular weight of the MCSP band observed in our samples appeared to be approximately ~300 kDa, which is higher than the expected molecular weight of ~240 kDa provided by the manufacturer. This discrepancy may be attributed to variations in sample preparation, differences in gel electrophoresis conditions, or post-translational modifications of the target protein. See supplementary file 5, Figure D for a positive control (LM cell lysate)

## Slide 3
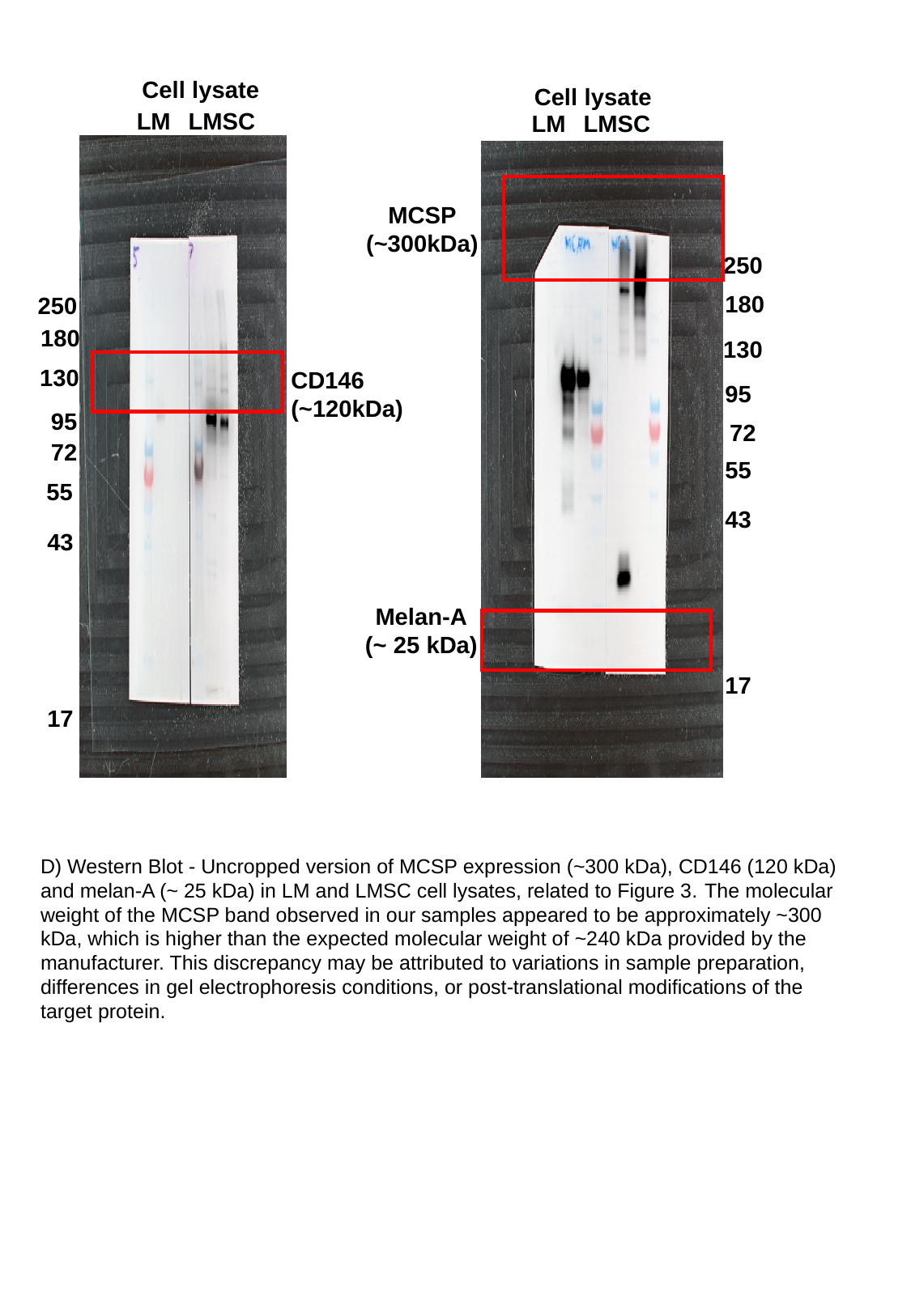

Cell lysate
Cell lysate
LM
LMSC
LM
LMSC
MCSP
(~300kDa)
250
180
250
180
130
130
CD146
(~120kDa)
95
95
72
72
55
55
43
43
Melan-A
(~ 25 kDa)
17
17
D) Western Blot - Uncropped version of MCSP expression (~300 kDa), CD146 (120 kDa) and melan-A (~ 25 kDa) in LM and LMSC cell lysates, related to Figure 3. The molecular weight of the MCSP band observed in our samples appeared to be approximately ~300 kDa, which is higher than the expected molecular weight of ~240 kDa provided by the manufacturer. This discrepancy may be attributed to variations in sample preparation, differences in gel electrophoresis conditions, or post-translational modifications of the target protein.

## Slide 4
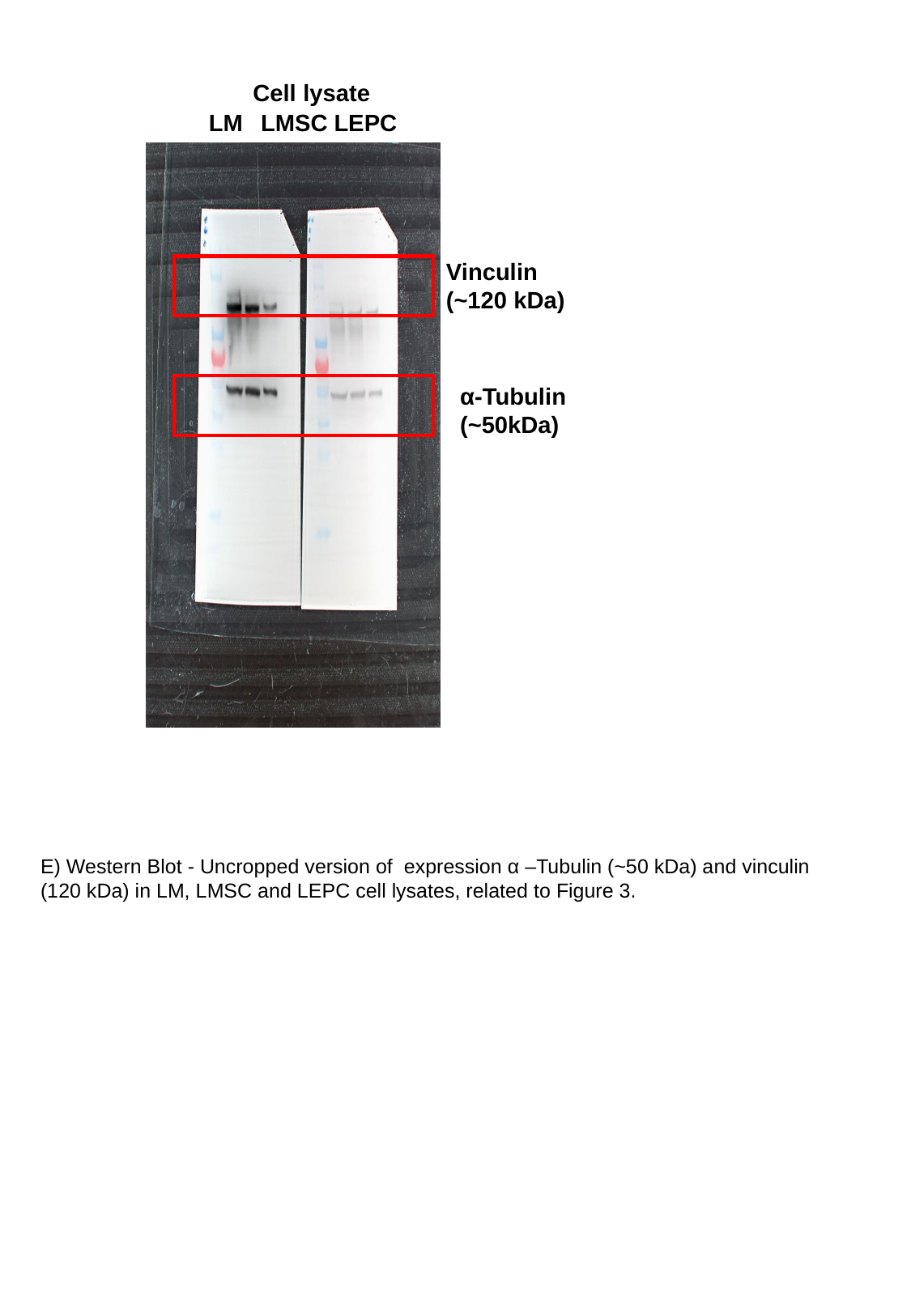

Cell lysate
LEPC
LM
LMSC
Vinculin
(~120 kDa)
α-Tubulin
(~50kDa)
E) Western Blot - Uncropped version of expression α –Tubulin (~50 kDa) and vinculin (120 kDa) in LM, LMSC and LEPC cell lysates, related to Figure 3.
