# Supplementary material for: Enrichment, Characterization, and Proteomic Profiling of Small Extracellular Vesicles Derived from Human Limbal Mesenchymal Stromal Cells and Melanocytes
Source: Cells. 2024 Apr 4;13(7):623. doi: 10.3390/cells13070623 (PMC11011788; doi:10.3390/cells13070623)
Supplement: Supplementary file 1 [file cells-13-00623-s001.zip › Supplementary File S6.pptx]

## Slide 1
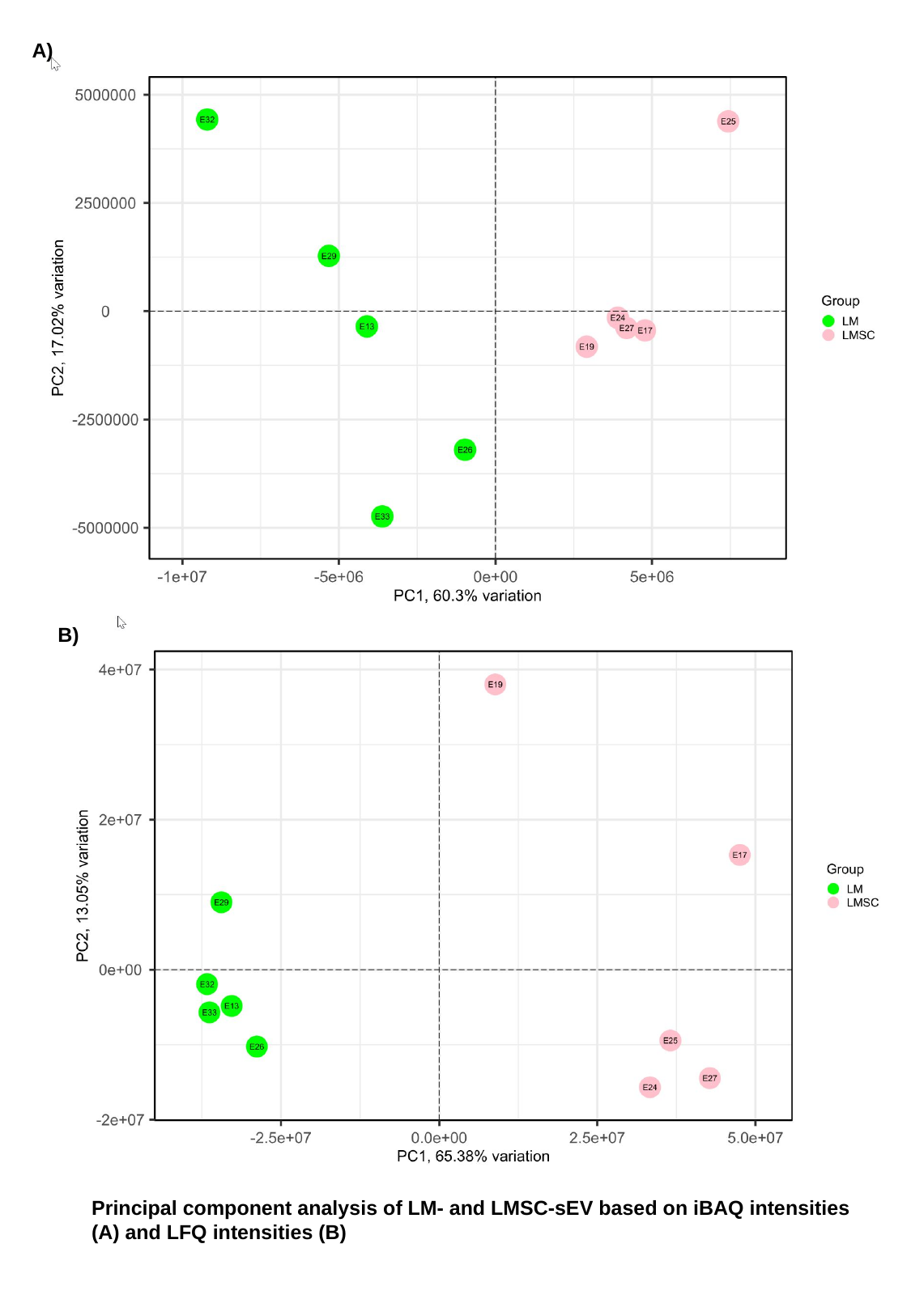

A)
B)
Principal component analysis of LM- and LMSC-sEV based on iBAQ intensities (A) and LFQ intensities (B)

## Slide 2
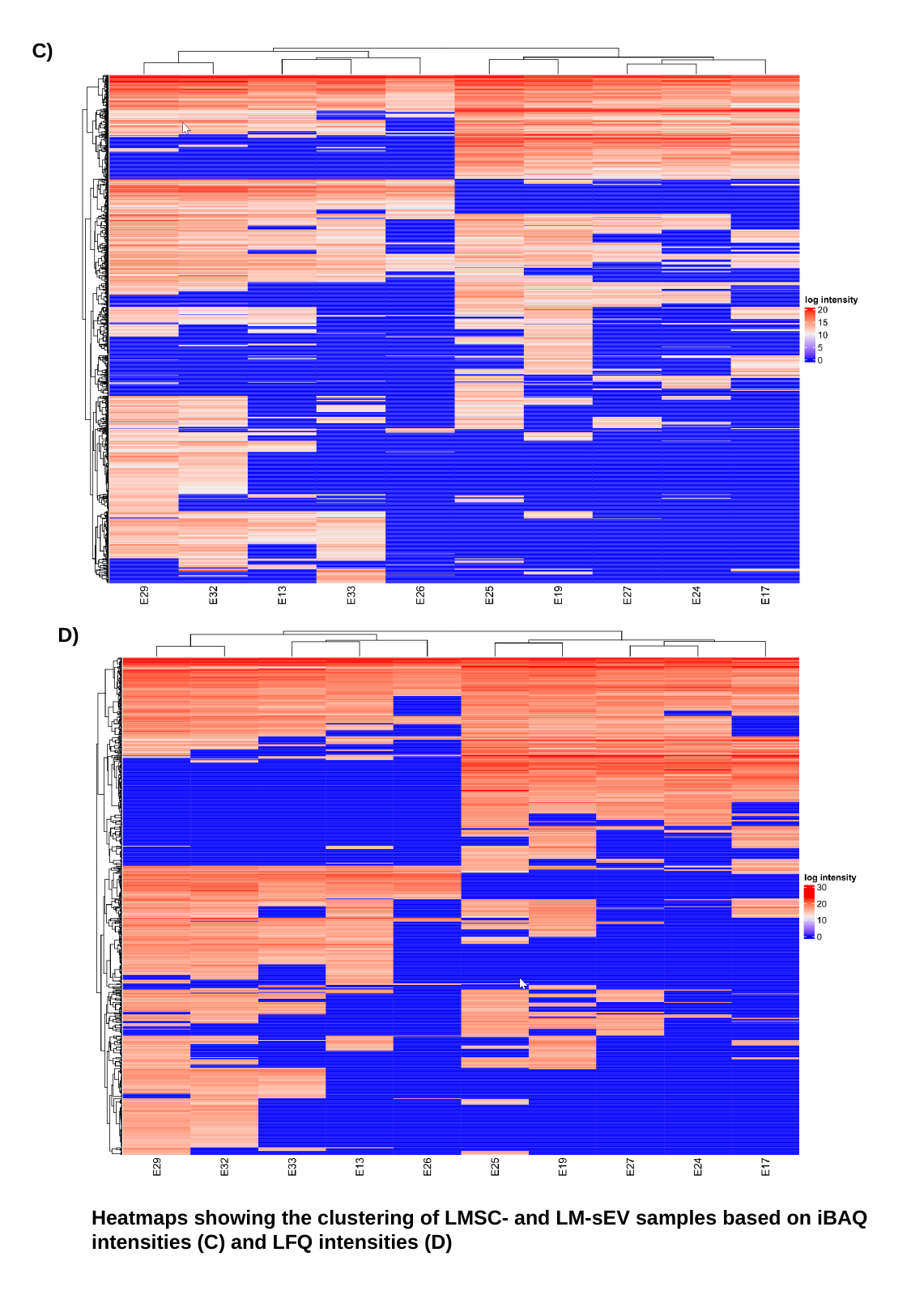

C)
D)
Heatmaps showing the clustering of LMSC- and LM-sEV samples based on iBAQ intensities (C) and LFQ intensities (D)
