# Supplementary material for: Enrichment, Characterization, and Proteomic Profiling of Small Extracellular Vesicles Derived from Human Limbal Mesenchymal Stromal Cells and Melanocytes
Source: Cells. 2024 Apr 4;13(7):623. doi: 10.3390/cells13070623 (PMC11011788; doi:10.3390/cells13070623)
Supplement: Supplementary file 1 [file cells-13-00623-s001.zip › Supplementary File S7.pptx]

## Slide 1
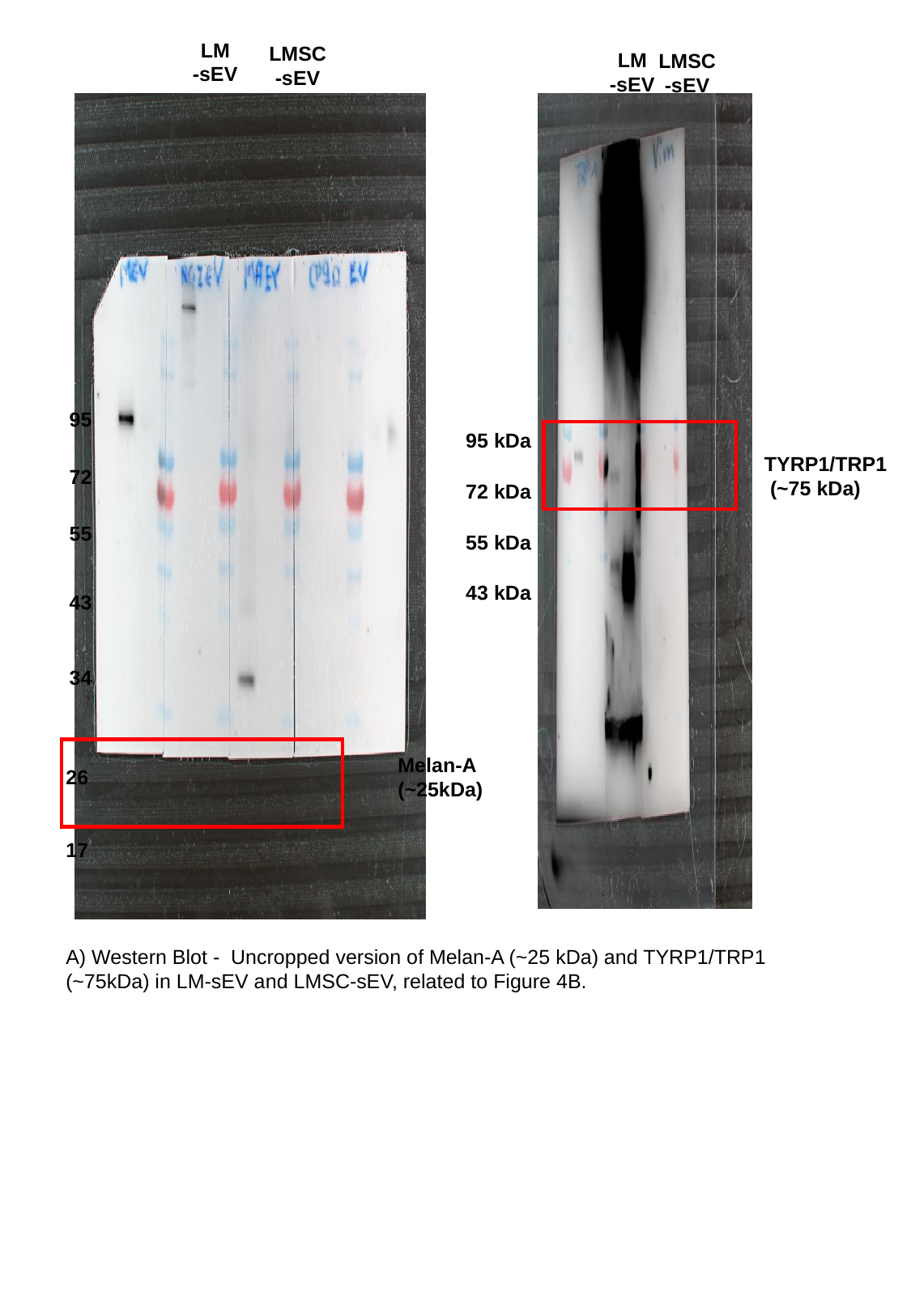

LM
-sEV
LMSC
-sEV
LM
-sEV
LMSC
-sEV
95
95 kDa
TYRP1/TRP1
 (~75 kDa)
72
72 kDa
55
55 kDa
43 kDa
43
34
Melan-A
(~25kDa)
26
17
A) Western Blot - Uncropped version of Melan-A (~25 kDa) and TYRP1/TRP1 (~75kDa) in LM-sEV and LMSC-sEV, related to Figure 4B.

## Slide 2
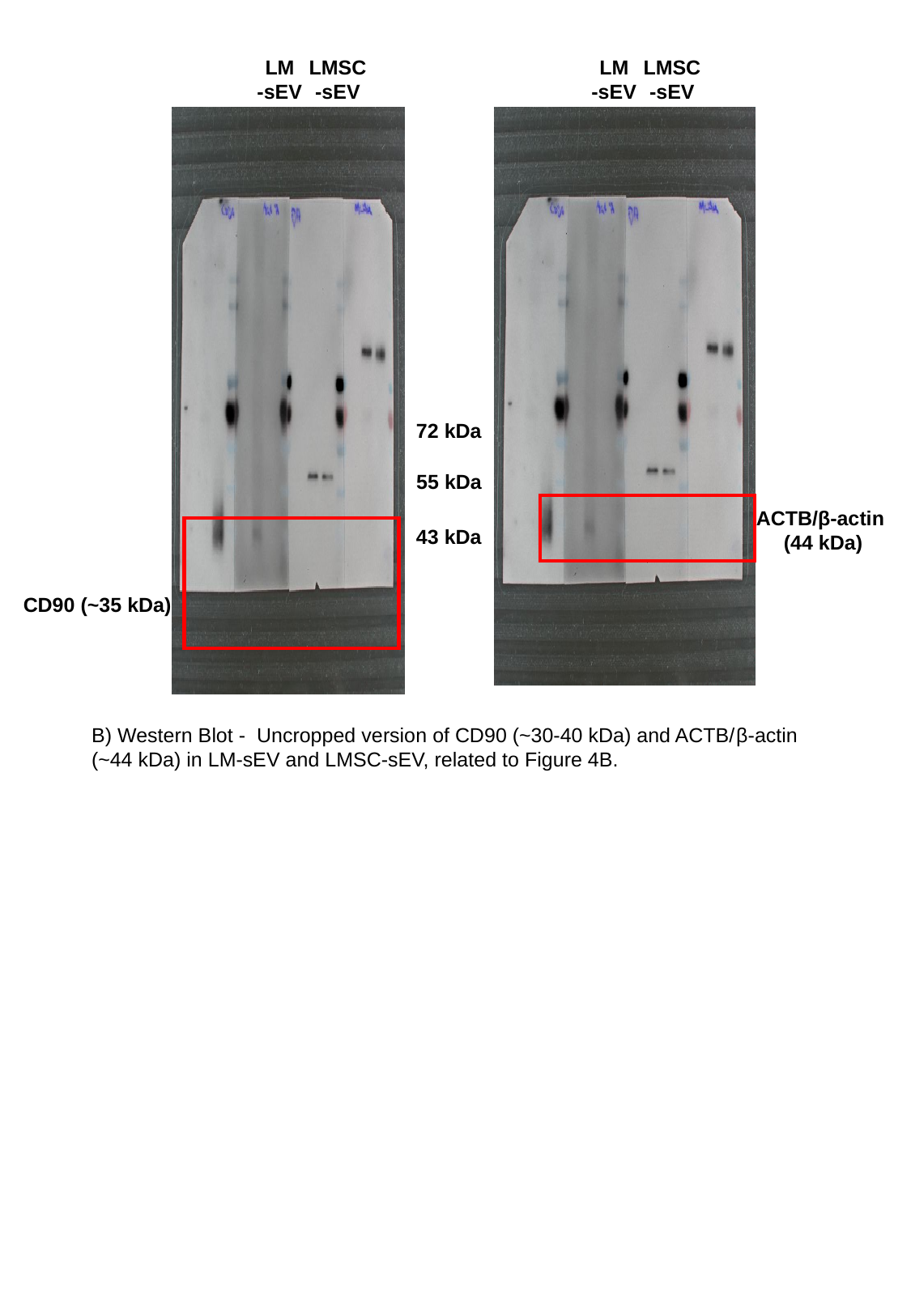

LMSC
-sEV
LMSC
-sEV
LM
-sEV
LM
-sEV
72 kDa
55 kDa
ACTB/β-actin
(44 kDa)
43 kDa
CD90 (~35 kDa)
B) Western Blot - Uncropped version of CD90 (~30-40 kDa) and ACTB/β-actin (~44 kDa) in LM-sEV and LMSC-sEV, related to Figure 4B.
