# Supplementary material for: Enrichment, Characterization, and Proteomic Profiling of Small Extracellular Vesicles Derived from Human Limbal Mesenchymal Stromal Cells and Melanocytes
Source: Cells. 2024 Apr 4;13(7):623. doi: 10.3390/cells13070623 (PMC11011788; doi:10.3390/cells13070623)
Supplement: Supplementary file 1 [file cells-13-00623-s001.zip › Supplementary File S8.pptx]

## Slide 1
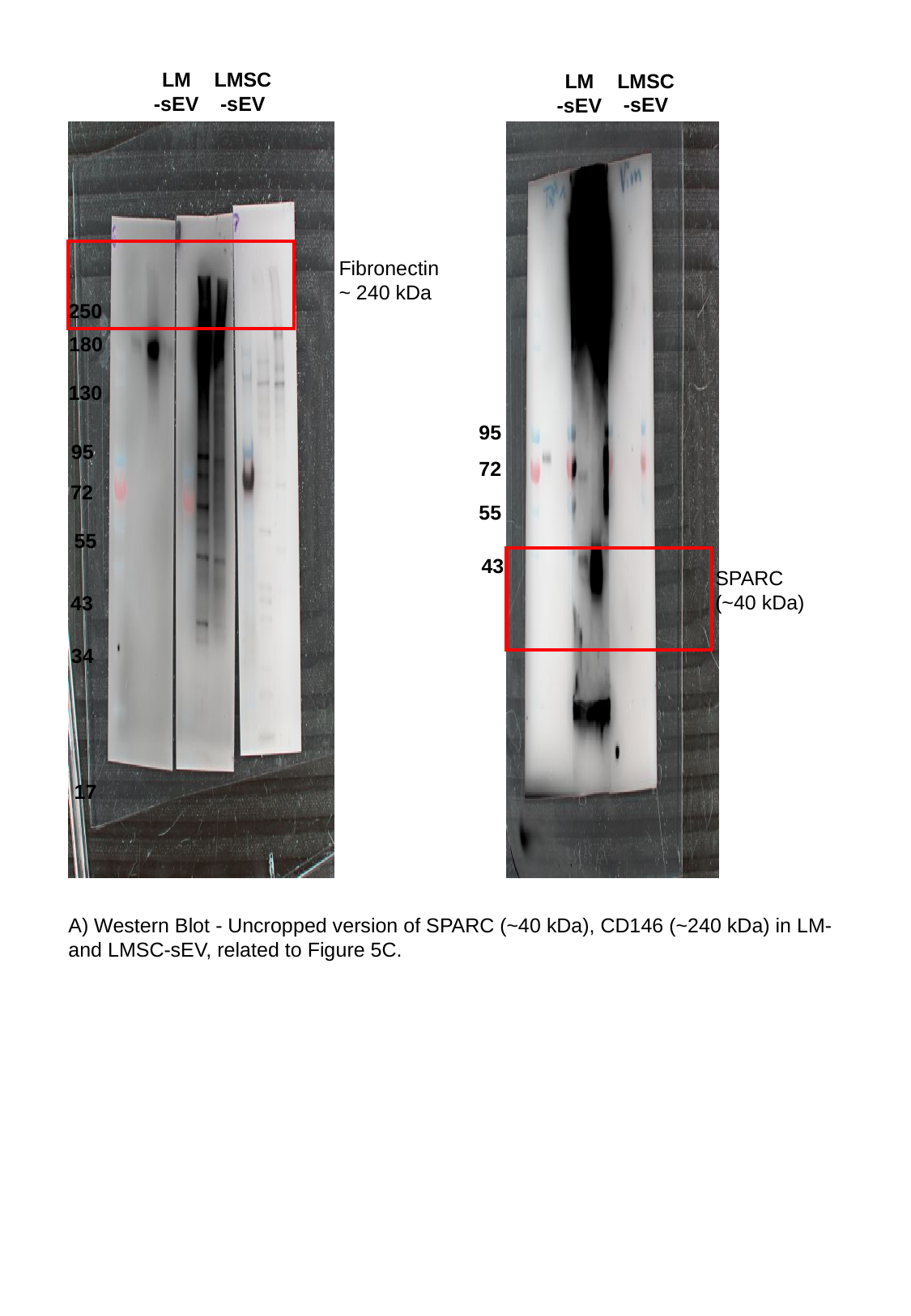

LMSC
-sEV
LM
-sEV
LMSC
-sEV
LM
-sEV
Fibronectin
~ 240 kDa
250
180
130
95
95
72
72
55
55
43
SPARC
(~40 kDa)
43
34
17
A) Western Blot - Uncropped version of SPARC (~40 kDa), CD146 (~240 kDa) in LM- and LMSC-sEV, related to Figure 5C.
