# Supplementary material for: Enrichment, Characterization, and Proteomic Profiling of Small Extracellular Vesicles Derived from Human Limbal Mesenchymal Stromal Cells and Melanocytes
Source: Cells. 2024 Apr 4;13(7):623. doi: 10.3390/cells13070623 (PMC11011788; doi:10.3390/cells13070623)
Supplement: Supplementary file 1 [file cells-13-00623-s001.zip › Supplementary Table S1.pdf]

Supplementary Table S1

**Tetraspanins**	CD9, CD63 and CD81
**Leukocyte-related markers**	CD1c, CD4, CD8,CD14, CD19, CD20, CD24, CD25 CD14,CD40, CD45, CD2, CD3, CD86
**Integrins**	CD11c (integrin αX), CD29 (Integrin β1), CD41b (integrin αIIβ), CD49e (integrin α5)
**Platelet and endothelial cell-related markers**	CD105, CD142, CD62P, CD42a
**MHC-associated antigens**	HLA-ABC and HLA-DPDQDR
**Stemness-related proteins**	SSEA-4, CD133/1, melanoma-chondroitin sulfate proteoglycan (MCSP), ROR1
**Immunogloublin-related cell adhesion molecules**	CD31, CD44, CD56, CD146, CD209, CD326
**Controls**	REA control, mIgG1
