# Supplementary material for: Enrichment, Characterization, and Proteomic Profiling of Small Extracellular Vesicles Derived from Human Limbal Mesenchymal Stromal Cells and Melanocytes
Source: Cells. 2024 Apr 4;13(7):623. doi: 10.3390/cells13070623 (PMC11011788; doi:10.3390/cells13070623)
Supplement: Supplementary file 1 [file cells-13-00623-s001.zip › Supplementary Table S8.pdf]

Supplementary Table S8: List of antibodies used in the study

Antibody (clone), Host species	Antibody dilution	Application	Antibody source	Catalog number
Alix (E6P9B) Rabbit mAb	1/1000	Western Blot	Cell Signaling Technology	92880S
CD63 (E1W3T) Rabbit mAb	1/1000	Western Blot	Cell Signaling Technology	52090S
CD9 (D3H4P) Rabbit mAb	1/1000	Western Blot	Cell Signaling Technology	13403S
CD81 (D3N2D) Rabbit mAb	1/1000	Western Blot	Cell Signaling Technology	56039S
Melan-A (EPR20380), rabbit mAb	1/1000	Western Blot	Abcam	ab210546
CD90/Thy1 (7E1B11), mouse	1/500	Western Blot	Novus Bio	NBP2-37330
Calnexin (C569) Rabbit mAb	1/1000	Western Blot	Cell Signaling Technology	2679S
BSA (D108Q) Rabbit mAb	1/1000	Western Blot	Cell Signaling Technology	23053S
NG2/CDPG4 (E3B3G) XP Rabbit mAb	1/1000	Western Blot Immunohistochemistry Immunocytochemistry	Cell Signaling Technology	43916S
CD146/ MCAM (4D8A9), Mouse	1:1000	Western Blot Immunohistochemistry Immunocytochemistry	Proteintech	66153-1-lg
MCAM (E3F3E), rabbit	1/1000	Western Blot	Cell Signaling Technology	81701
TRP1 (EPR21960), rabbit	1/1000	Western Blot	Abcam	ab235447
Fibronectin (IST-4) mouse	1/250	Western Blot	Merck/ Sigma Aldrich	F0916
ß-Actin (D6A8) Rabbit mAb	1/1000	Western Blot	Cell Signaling Technology	8457S
SPARC		Western Blot		
CD117, PE (YB5.B8), Mouse	5µl/10^6 cells	Flow Cytometry	BD Biosciences	561682
CD90, APC (5E10), Mouse	5µl/10^6 cells	Flow Cytometry	BD Biosciences	559869
Anti-Mouse IgG, Horseradish Peroxidase (Polyclonal), Goat	1/10000	Western Blot	Jackson ImmunoResearch Labs	115-035-003
Anti-Rabbit IgG, Horseradish Peroxidase (Polyclonal), Goat	1/10000	Western Blot	Jackson ImmunoResearch Labs	111-035-003
Gp100 (HMB45), rabbit	1:100	Immunohistochemistry	Abcam	Ab787

Vimentin (280618), rat	1:500	Immunohistochemistry	R&D systems	MAB2105-SP
